# Effect of different application strategies of universal adhesive used for immediate and delayed dentin sealing on the microtensile bond strength of self-adhesive resin cement to dentin with and without aging

**DOI:** 10.4317/jced.60202

**Published:** 2023-03-01

**Authors:** Soodabeh Kimyai, Mahmoud Bahari, Mahdi Abed-Kahnamouei, Mohammad-Esmaeel Ebrahimi-Chaharom, Mahdi-Hoseini Asl-Oskouei

**Affiliations:** 1 Professor, Department of Operative Dentistry, Faculty of Dentistry, Tabriz University of Medical Sciences, Tabriz, Iran; 2 Assistant Professor, Dental and Periodontal Research Center, Tabriz University of Medical Sciences, Tabriz, Iran; 3 Associate Professor, Department of Operative Dentistry, Faculty of Dentistry, Tabriz University of Medical Sciences, Tabriz, Iran; 4 General Practitioner, Department of Operative Dentistry, Faculty of Dentistry, Tabriz University of Medical Sciences, Tabriz, Iran

## Abstract

**Background:**

Bond strength of indirect restorations is a very important issue that should be given special attention. Immediate dentin sealing (IDS) technique has been suggested in recent years. The aim of the present study was to investigate the effect of different strategies of universal adhesive application for immediate and delayed dentin sealing (DDS) on the microtensile bond strength (μTBS) of self-adhesive resin cement with and without aging.

**Material and Methods:**

In this experimental study, 24 healthy human third molars were selected. After exposing the occlusal dentin, the teeth were randomly divided into two groups of 12 based on the All-Bond Universal adhesive application strategy (etch-and-rinse or self-etch). Each group was further subdivided into two (n=6) based on IDS or DDS technique. Then composite blocks were cemented on the occlusal surface with self-adhesive resin cement. After cutting the samples into 1 mm2 cross-sections, half of the samples of each subgroup were subjected to µTBS test after one week, and the other half were tested under μTBS after 10,000 thermal cycles. Data were analyzed using three-way ANOVA (*P*<0.05).

**Results:**

μTBS was significantly affected by all three factors of bond strategy, sealing technique and aging. There was also a significant interaction between the three factors.

**Conclusions:**

Immediate dentin sealing improved μTBS. The etch-and-rinse strategy resulted in higher μTBS, while aging led to a decrease in μTBS.

** Key words:**Adhesives, dental bonding, dentin, universal, sealing.

## Introduction

Nowadays esthetics is one of the most basic demands of patients visiting dental offices. Indirect restorations can fulfill most of these needs. Tooth preparation for indirect restorations, removes a larger surface of the tooth, and this increases the probability of dentinal tubules exposure. Because the highest degree of dentin permeability is immediately after preparation, dentin management is critical at this stage. The penetration of bacteria and saliva causes the colonization of microorganisms and sensitivity after treatment, all of which can be potential stimuli for the pulp (1). Because of this problem, the use of adhesives on freshly cut dentin was suggested by Magne *et al*., for the first time, which is known as the immediate dentin sealing (IDS) technique. In this method, the adhesive is applied to dentin immediately after the tooth preparation and before impression. This technique is based on the fact that freshly cut dentin has the maximum permeability and this type of dentin is the most ideal type for bonding (2). Furthermore, the use of the IDS technique prevents the penetration of microorganisms and saliva into the dentinal tubules and protects the pulp from infectious and thermal factors. IDS technique increases the bond strength of resin cements under indirect restorations. Moreover, this technique increases the success of treatment in the long term by reducing microleakage. A clinical investigation performed on vital teeth prepared for indirect restorations showed that in a period of 24 months, the sensitivity after treatment in the IDS group was significantly lower than the control group (3).

Various adhesive systems have been used for IDS over time. Magne *et al*., demonstrated that in the case of using 3-step etch-and-rinse (E&R) adhesive system, the bond strength increases significantly in the IDS technique compared to the delayed sealing (4). Ferreira-Filho *et al*., showed that after seven days of water storage, the groups with IDS had higher μTBS than the control group, although XP Bond and Clearfil SE Bond did not have significant differences. However, after three months of water storage, IDS groups did not differ significantly from control group (DDS) (5).

Recently, universal or multi-mode adhesives have been introduced. These adhesives made bonding steps easier and could be used with selective etching, E&R and self-etch (SE) strategies. These adhesives can be adhered to ceramics, indirect composite resins and metal alloys (6-8). Perdigão *et al*., showed that universal adhesives have a higher bond strength than two-step SE adhesives (6), while in the study of Vermelho *et al*., there was not a significant difference between the bond strength of universal adhesives and 3-step E&R or 2-step SE adhesives (7). Conversely, Muñoz *et al*., reported that universal adhesives have lower bond strength than two-step SE and two-step E&R adhesives (8). Furthermore, it has been concluded that there was no significant difference in μTBS of universal adhesives between E&R and SE application strategies (9).

Considering that universal adhesives have a different basics from the previous generations and the characteristics of these types of adhesives are different, and so far no study has been conducted on their use in relation to the IDS technique, the present study was performed to investigate the effect of different strategies of universal adhesive application for IDS and DDS on the μTBS of self-adhesive resin cement with and without aging.

## Material and Methods

24 healthy human third molar teeth were used in this experimental study. The teeth were free of caries, cracks, fractures and structural defects in the visual examination and under a stereomicroscope (Nikon, SMZ1000, Tokyo, Japan). The study protocol was approved by the Regional Medical Research Ethics Committee. The teeth were placed in a 0.5% chloramine-T bacteriostatic/bactericidal solution (Merck KGaA, Darmstadt, Germany) for 7 days and then stored in distilled water in a refrigerator at 4ºC, with renewal of the storage medium regularly. At a 24-hour interval before the initiation of the procedural steps of the study, the teeth were transferred into distilled water at 23±2ºC for conditioning.

**Figure 1:**
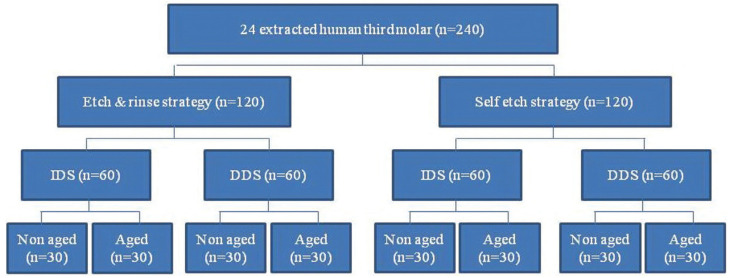
Schematic presentation of study groups and subgroups.

The occlusal enamel was removed by diamond saw under constant water spray and the dentin exposed. Then, the occlusal dentin was polished with 600-grit silicon carbide sandpaper (3M of Brazil, Sumaré, SP, Brazil) under running water for standardization of smear layer. The roots of the teeth were mounted in acrylic resin blocks up to 2 mm below the CEJ. Then, the teeth were randomly divided into two groups of 12 based on the strategy of universal adhesive application (E&R or SE). Each group was further subdivided into two (n=6) based on performing IDS or DDS (Fig. 1).

In the E&R group, the E&R strategy was used for All-Bond Universal (ABU) adhesive (Bisco Inc., Schaumburg, IL, USA) immediately after occlusal dentin preparation (IDS Technique). The dentin surface was etched with 35% phosphoric acid gel (Bisco Inc., Schaumburg, IL, USA) for 15 seconds, then was rinsed with water for 15 seconds, and gently dried with cotton pellets, so that only excess moisture was removed. Then, two layers of bonding were applied to the dentin surface for 10-15 seconds with a rubbing motion using a microbrush. Gentle air was blown over the surface for 10 seconds to evaporate the bonding solvent. Finally, light irradiation was done for 10 seconds with an intensity of 1200 mW/cm2 by a LED light curing device (Dentamerica Inc., City of Industry, CA, USA). Glycerin gel (KY gel, Johnson & Johnson do Brasil, Sao Paulo, Brazil) was used to cover the surface, so that oxygen could not hinder surface polymerization, and then it was polymerized again for 20 seconds. After removing this gel, cementing was done immediately after bonding application for half of specimens (IDS subgroup) and the other half of the samples were kept for 7 days in an incubator at 37°C before cementing the composite blocks (DDS subgroup). Composite blocks were prepared from shade A2 of universal microhybrid composite resin (Spectrum, Dentsply Sirona, Konstanz, Germany) with a thickness of 4 mm and a diameter of 12 mm in a silicone mold. One side of the composite blocks were abraded with a 600-grit silicon carbide disc with water spray to produce a smooth surface of standard roughness for cementation. Then, the composite surface was abraded for 10 seconds with 50-micron aluminum oxide particles by air-abrasion device (Microblaster Dento-Prep TM, Dental Microblaster, Denmark) with a distance of 5 mm from the surface. For the cementing process, at first, the surface of the composite block was first cleaned with distilled water in an ultrasonic device for 10 minutes and then it was dried. Then, silane (Silane Bond Enhancer, Pulpdent Corporation, Watertown, MA 02472, USA) was applied on the composite surface. Next, the composite blocks were cemented using self-adhesive resin cement (Bifix SE, Voco Gmbh, Cuxhaven, Germany). The samples were light cured from the buccal, lingual and occlusal surfaces for 40 seconds.

In the SE group, a similar procedure was performed, with the difference that instead of E&R strategy, SE strategy was used for ABU adhesive application. So, etching was not used as separate step, and ABU was applied according to the manufacturer’s instructions for SE strategy and cured for 10 seconds. All procedures were performed by a single operator.

For preparing the samples for the µTBS test, the tooth-composite block assemblies were cut vertically into rod-shaped samples with a surface area of 1 mm2. 10 samples were obtained from each tooth. Half of the samples from each subgroup were randomly selected and μTBS was performed after 7 days of storage in an incubator, and μTBS was measured in the remaining half of each subgroup after 10,000 times of thermocycling at a temperature of 5±5°C/55±5°C. The µTBS test was performed by a microtensile tester (Bisco Inc., Schaumburg, IL, USA) at a loading speed of 0.5 mm/min and the data was recorded in Newton.

Three-way ANOVA test was used to investigate the effect of the three factors of bond strategy, sealing technique and aging on the µTBS. In each of the IDS and DDS subgroups, Independent Samples T-Test was used to investigate the effect of aging on the μTBS. In each bond strategy, to investigate the effect of sealing technique on the μTBS of subgroups with and without aging two-way ANOVA was used. The significance level was set at *P*<0.05.

## Results

**Figure 2:**
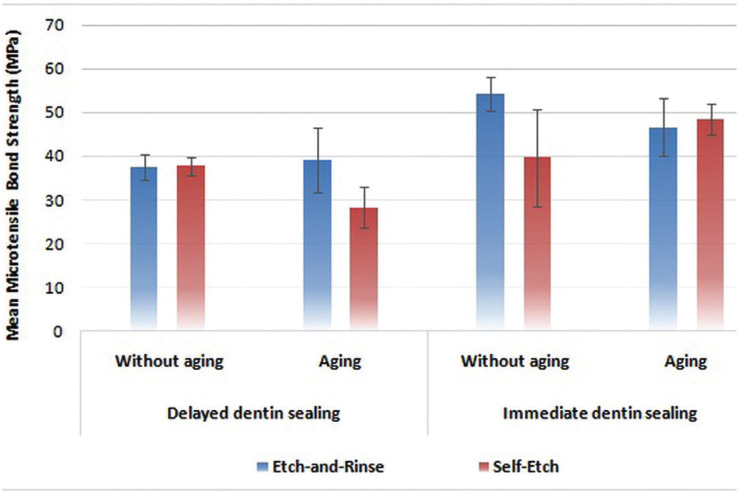
Bar graph of the mean μTBS values in the study groups and subgroups.

Table 1 and Figure 2 show means and standard deviations related to μTBS of study groups and subgroups. Three-way ANOVA showed that all three factors of bond strategy (*P*<0.001), sealing technique (*P*<0.001) and aging (*P*=0.04) had significant effects on the μTBS. Hence, mean µTBS in the E&R strategy was higher than the SE strategy. Also, the mean μTBS in the IDS was higher than the DDS, and the mean μTBS in the aged subgroup was lower than the mean in the non-aged subgroup.

**Table 1:**
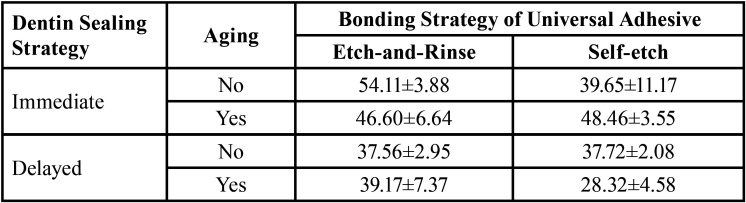
Means and standard deviations of the μTBS values (MPa) in the study groups and subgroups.

There was no significant interaction between bond strategy and sealing techniques (*P*=0.559), nor between bond strategy and aging (*P*=0.104), while there was an interaction between sealing technique and aging (*P*=0.006). In other words, the effect of aging on the μTBS was not the same in IDS and DDS methods. The results of Independent Samples T-Test showed that in the DDS, there was a statistically significant difference between the µTBS variable in the aged and non-age subgroups (*P*=0.001) and the µTBS in the non-aged subgroup was higher than in the aged subgroup. However, in the IDS, there was no statistically significant difference between the μTBS in the aged and non-aged subgroups (*P*=0.54). There was a significant interaction between bond strategy, sealing technique and aging (*P*<0.001), also. In other words, the effect of aging on the μTBS was not the same in IDS and DDS technique and in the two bond strategies.

For each of the bond strategies, two-way ANOVA was used to investigate the effect of sealing technique on the μTBS in aged and non-aged subgroups. The results of two-way ANOVA showed that in both of bond strategies, the sealing technique had a significant effect on μTBS (*P*<0.001). The μTBS in the IDS was higher than that in the DDS. Also, in both bond strategies, there was an interaction between sealing technique and aging (*P*<0.001). In the E&R strategy, aging significantly affected μTBS (*P*=0.007) and led to a decrease in μTBS. While in the SE strategy, aging did not lead to a difference in the μTBS (*P*=0.80).

## Discussion

IDS has been proposed in order to provide several benefits of improvement of bond quality, increased sensitivity of dentin, gap formation and bacterial microleakage (10). Different adhesive generations have a different basics and characteristics from each other. IDS technique using different adhesive generations has been investigated in several studies owing to the importance of the issue (2,3,5,11,12). Universal or multi-mode adhesives have been introduced into the market recently (13). Little is known about the efficacy of IDS technique using universal adhesive systems. Therefore, in the present study IDS technique was evaluated using the ultramild universal adhesive system applied with two different bonding strategies (E&R vs SE).

The results of the present study showed that in the IDS technique, μTBS was significantly higher than the DDS, which is in line with the results of previous studies (3,4,10-12,14). Breemer *et al*., reported that IDS technique using 3-step E&R adhesive system (Optibond FL) resulted higher μTBS compared to DDS technique (12). Hardan *et al*., reported that regardless of bond strategy, IDS improves bond strength (15). It has been shown that applying adhesive on the dentin immediately after tooth preparation can lead to an increase in bond strength (16). It has also been reported that in the usual DDS technique, a gap is formed between resin and dentin (17). However, the use of IDS leads to the reduction of microleakage between dentin and restoration, reduction of bacterial contamination, sensitivity of dentin and finally better adaptation of restoration (2,18,19). Another reason for the higher bond strength in the IDS can be related to the fact that the first dentin hybridization occurs in stress-free conditions (11). Contrary to the results of the present study, Ferreira-Filho *et al*., found no difference in μTBS between the IDS and DDS techniques after 3 months of water storage when performed with 4th, 5th, 6th and 7th generations of adhesive systems (5). It seems that the reason for the difference in the results of the above study and the present study is the use of different adhesives. It has been reported that the ability of the adhesive to form a hydrophobic resin coating as well as the amount of adhesive filler can be effective in the results of the IDS (16).

As another finding, present study demonstrated that the μTBS in the E&R strategy of ABU was significantly higher than the SE strategy, which was in line with previous studies (20,21). It seems that the reason for higher µTBS in the E&R strategy is the formation of resin tags. It has been reported that in ultra-mild adhesives like ABU resin tags are not formed in self-etch strategy. While in the E&R strategy, resin tags are formed, which leads to the provision of micromechanical retention due to the good hybridization of the dentin and finally the improvement of the bond (20). Another reason can be related to the removal of the smear layer after the acid etching process and the better penetration of monomers into the dentin (13). In contrary to the result of the present study, Da Rosa *et al*., reported no difference in the μTBS of universal adhesive in the E&R and SE strategies (22). The reason for this can be related to the difference in the type and composition of the adhesives used. Because in that study, the adhesives were mild universal adhesives, while the adhesive used in the current study was ultra-mild. Mild adhesives are more acidic than ultra-mild adhesives, and in the case of SE strategy, they are able to remove the smear layer better and, as a result, monomers penetrate better into the dentin. Therefore, the bond strength has no difference with the E&R strategy, in which 32-37% phosphoric acid with pH=0.1-0.4 is used to remove the smear layer (21). But in ultra-mild adhesives such as ABU, the pH of the adhesive is higher, and as a result, due to the lower acidity, the removal of the smear layer and the formation of the resin tags are not complete. Furthermore, Yamauchi *et al*., found no difference between the bond strength of E&R and SE strategies in universal adhesives. The reason for the contradictory results with the present study can be due to the difference in the bond strength measurement test. In the previous research, the modified shear bond strength test was used. It has been reported that the type of bond strength test can affect the results. It has been reported that phosphoric acid etching leads to a significant decrease in free energy and dentin parameters such as wettability and degree of polymerization and does not improve the bond (23). Also, in another study, no difference was reported between the 24-hour μTBS of universal adhesives in E&R and SE strategies. It seems that the reason for this difference is related to the difference in composition, acidity and solvents of different universal adhesives. It has been reported that in the SE strategy, solvents such as acetone compared to ethanol can play a more effective role in preventing collagen degradation in the hybrid layer because of better water evaporation. The presence of water can lead to hydrolysis of resin and enzymatic breakdown of collagen fibrils (24).

Furthermore, in the present study, it was found out that μTBS in the aged subgroup was significantly lower than the non-aged subgroup, which was in line with the results of previous studies (23,24). It seems that the reason for the decrease in bond strength after thermocycling is thermal stress and some plasticization at the dentin-adhesive interface. It has been reported that this can lead to changes in mechanical properties (13,25). Also, hot water can accelerate the hydrolysis of unprotected collagens and separate resin oligomers (26). Contrary to the results of the present study, Pashaev *et al*., reported that there was no difference between μTBS of ABU adhesive either in the SE strategy or in the E&R strategy after aging (water storage for 6 months). Different aging processes might be an explanation for the different results between the above-mentioned study and the present study (27). Yao *et al*., reported that 10,000 thermal cycles is equivalent to one year of physiological aging (28). Furthermore, contrary to our findings, Wagner *et al*., did not find any difference between bond strength of universal adhesive before and after thermocycling (29). It seems that the difference in the aging protocol is the reason for the difference in the results of these studies. In the above-mentioned study, 5000 thermal cycles were used for aging purposes.

Another finding of the present study was that aging did not have the same effect on the μTBS of universal adhesive in IDS and DDS techniques. So that in the IDS, the μTBS did not decrease after aging, while in the DDS, the bond strength decreased after aging. In this regard, Hardan *et al*., reported that the IDS is more resistant to mechanical loading and thermocycling for a longer period of time compared to the DDS (15).

Also, the current study demonstrated that aging did not have the same effect on μTBS of ABU in IDS and DDS performed with either E&R or SE strategies. Thus, in the SE strategy, aging did not lead to a difference in μTBS. While in the E&R strategy, aging led to a decrease in μTBS. In the E&R strategy, this can be caused by the bond decomposition following the aging process. It has been reported that following aging in the E&R strategy, resin washing and collagen degradation occur in the hybrid layer (30). In this regard, it has been reported that over time, in the E&R strategy of universal adhesives, the breakdown of the hybrid layer happens within 6 months to 3 to 5 years (22). It has also been reported that the presence of exposed collagen fibrils after acid etching can increase the activity of endogenous enzymes such as matrix metalloproteinases and cysteine cathepsins and lead to accelerated degradation of the hybrid layer (31). Contrary to the results of the present study, Zhang *et al*., reported that in ABU adhesive, after 12 months of aging (keeping the samples in NaCl/NaN3 solution at 37°C), μTBS decreased in the SE strategy. Those researchers stated that the nanoleakage that was observed in the 24-hour evaluation of the samples could justify the breakdown of the hybrid layer in some universal adhesives in the SE strategy (24). Also, contrary to the present research, Cuevas-Suarez *et al*., demonstrated that in mild universal adhesives, aging did not lead to a difference in µTBS either in the SE or in the E&R strategy, and the bond strength remained constant over time regardless of the strategy used (20). It seems that the reason for the difference is related to the difference in the composition and acidity of different universal adhesives.

Considering the laboratory nature of the present study and taking into account the fact that in clinical conditions, factors such as masticatory stress, pH, saliva and moist oral environment can be effective in the faster decomposition of the bonding interface, it is suggested that further studies be performed with more simulation of the oral environment, including the investigation of other methods of aging and load cycling. Since it has been shown that the pH of universal adhesives can affect their performance it is suggested to investigate other universal adhesives with different acidity in future studies (20). Moreover, it is suggested to investigate the adhesive interface microstructure with an electron microscope.

## Conclusions

According to the limitations of the present study, it can be concluded that IDS with ABU adhesive improved μTBS. The E&R strategy led to higher μTBS for ABU than the SE strategy. Furthermore, aging led to the significant decrease of μTBS.
